# Normobaric Hyperoxia (NBHO): An Adjunctive Therapy to Cerebrovascular Recanalization in Ischemic Stroke

**DOI:** 10.14336/AD.2023.0227

**Published:** 2023-10-01

**Authors:** Wenbo Hu, Weili Li, Ruchi Mangal, Milan Jia, Xunming Ji, Yuchuan Ding

**Affiliations:** ^1^Department of Neurology, Xuanwu Hospital, Capital Medical University, Beijing, China.; ^2^Cerebrovascular Diseases Research Institute, Xuanwu Hospital, Capital Medical University, Beijing, China.; ^3^Beijing Institute of Brain Disorders, Capital Medical University, Beijing, China.; ^4^Department of Neurosurgery, Wayne State University School of Medicine, Michigan, USA.; ^5^Beijing Key Laboratory of Hypoxic Conditioning Translational Medicine, Xuanwu Hospital, Capital Medical University, Beijing, China.; ^6^John D. Dingell VA Medical Center, Detroit, Michigan, USA.

**Keywords:** normobaric hyperoxia, ischemic stroke, recanalization, neuroprotective

## Abstract

Acute ischemic stroke (AIS) is a serious neurological disease. Normobaric hyperoxia (NBHO) is both a non-invasive and easy method that seems to be able to improve outcomes after cerebral ischemia/reperfusion. In clinical trials, normal low-flow oxygen has been shown to be ineffective, but NBHO has been shown to have a transient brain-protective effect. Today, NBHO combined with recanalization is the best treatment available. NBHO combined with thrombolysis is considered to improve neurological scores and long-term outcomes. Large randomized controlled trials (RCTs), however, are still needed to determine the role they will have in stroke treatment. RCTs of NBHO combined with thrombectomy have both improved infarct volume at 24 hours and the long-term prognosis. These two mechanisms most likely play key roles in the neuroprotective actions of NBHO after recanalization, including the increase in penumbra oxygen supply and the integrity of the blood-brain barrier (BBB). Considering the mechanism of action of NBHO, oxygen should be given as early as possible to increase the duration of oxygen therapy before recanalization is initiated. NBHO can further prolong the existence time of penumbra, so that more patients may benefit from it. Overall, however, recanalization therapy is still essential.

Stroke is the leading cause of functional disability and mortality worldwide. Ischemic stroke accounts for 80% of all stroke types. Therefore, the treatment of ischemic stroke is essential. Although intravenous thrombolysis and intravascular mechanical thrombectomy have been proven effective and are the current recommended treatments, clinical outcomes are worse than expected. This is despite successful endovascular treatment (EVT), indicating the need for a novel adjuvant therapy to improve brain protection and reduce treatment complications [1-3]. Currently, oxygen therapy is becoming more accepted. Normobaric hyperoxia (NBHO) is considered safe and easy to use regardless of location, including in an ambulance, emergency room, or operating room, which is valuable for the acute phase of "time is brain" [4, 5].

NBHO commonly refers to administering 40-100% oxygen continuously to patients under normal atmospheric pressure. In clinical trials, proper usage of NBHO has been correlated with efficacy outcomes. Compared with normobaric low-flow inhalation, which was proven ineffective, NBHO seems to have a clear neuroprotective effect. Brain damage after ischemic stroke can be ameliorated by rescuing the "ischemic penumbra," which is the severely hypoperfused and hypoxic, electrically silent brain tissue. NBHO can freeze the penumbra and prevent the ischemic core from growing, leading to improved neurological outcomes within 24 hours [6-8]. However, NBHO alone could not improve long-term outcomes in most clinical studies [7]. This can be attributed to infarction continuing to grow due to the lack of recanalization. When NBHO was applied with a vascular recanalization strategy, the neurological outcome and prognosis of AIS patients were both improved.

## NBHO combined with Reperfusion Strategy

### Intravenous thrombolysis

In 2017, Shi et al. conducted a clinical study that not only used NBHO in combination with intravenous thrombolysis but also included biomarker analysis and neurological function score assessment. Patients with anterior circulation stroke (<4.5 hours) were randomly assigned to either oxygen therapy (10 L/min) or room air for 4 hours. The study found that NBHO therapy could reduce peripheral blood occludins levels and protect the BBB in AIS patients. NBHO could also improve the outcomes of AIS patients who have already been treated with intravenous tPA thrombolysis [9]. However, the sample size of this study was small, and the long-term prognosis of these patients was not tracked.

In 2021, Li et al. conducted an observational study that included patients with the same criteria and treatment intervention. The multivariate logistic regression analysis showed that NBHO combined with intravenous thrombolysis benefited from 90-day functional independence [10]. Although this study analyzed the long-term prognosis, a large RCT is still necessary to provide high-level evidence (Table 1).

**Table 1:** NBHO combined with Reperfusion Strategy. Experiments (NBHO + intravenous thrombolysis) were filled in purple, while experiments (NBHO+EVT) were filled in blank.

Study Sponsor	Year	Study Type	Sample size	Type of patients	NBHO treatment and duration	Outcome	Efficacy
Shi et al.	2017	Cohort Stufy	18	Patients with acute ischemic stroke	Oxygen, 10L/min, 4 hours, by facemask	Blood Occludin and Claudin-5 Levels (baseline-24hours-72 h) NIHSS (1.3.7days)	Beneificial
Li na	2021	Observational study	227 (125+102)	125 patients received NBHO therapy combined with intravenous thrombolysis, while 102 patients received intravenous thrombolysis only.	Oxygen, 10L/min, 4 hours, by facemask	mRS at 90 days	Beneificial
Cheng et al.	2021	Prospective RCT	180 (91+89)	stroke patients with large vessel occlusion	high-flow NBHO by a Venturi mask (FiO_2_ 50%, flow 15 L/min) Starting After vessel recanalization for 6 h	mRS at 90 days	Beneificial
Poli et al.	2017	Single-blind RCT	456	Patients with acute ischemic stroke due to large vessel occlusion likely to receive endovascular mechanical thrombectomy	100% oxygen, ≥ 40 L/min, via a sealed non-rebreather facemask with reservoir	Ischemic core growth from baseline to 24 hours	On going
Li et al.	2018	Single-blind RCT	86	Patients with acute ischemic stroke who received endovascular treatment	Oxygen, 10L/min, via oxygen storage facemask	Cerebral infarct volume evaluated through MRI (DWI), 24-48 h after randomization	Beneificial
Li et al.	2021	triple-blind RCT	280	patients with indications for endovascular thrombectomy, who randomized<6 hours after stroke onset	100% oxygen (≤30min after admission), 10L/min, 4 hours, by a sealed non-ventilating oxygen storage mask	mRS at 90 days	On going

Abbreviations: NBHO, Normobaric Hyperoxia; NIHSS, the National Institute of Health Scale Score; RCT, Randomized Clinical Trial; mRS, modified Rankin Score; MRI, Magnetic Resonance Imaging; DWI, Diffusion Weighted Imaging.

### Endovascular thrombectomy (EVT)

The European PROOF study by Poli et. al. included 460 AIS patients (<3hours) with anterior circulation macrovascular occlusion. The oxygen inhalation group received mask oxygen inhalation of ≥ 40 L/min 100% concentration. However, NBHO cannot reduce the volume progression of the infarction on an MRI in 24 hours [11]. This may be because rapid recanalization will lead to a short treatment time of NBHO, resulting in the "freezing time" of the penumbra not being long enough to manifest its effect.

Another RCT conducted by Cheng et. al. followed 233 patients receiving endovascular treatment with large vessel occlusion in their anterior circulation. They were given either 6 hours of high-flow Venturi mask oxygen therapy (FiO2 50% 15 L/min) or conventional low-flow nasal catheter oxygen support therapy (3 L/min) immediately after recanalization. This study found that NBHO can improve functional prognosis, decrease mortality, and reduce the infarct volume of AIS patients [12].

In the OPENS-1 study by Li et. al., 86 patients who had anterior circulation macrovascular occlusive stroke with an indication for interventional therapy were randomly selected within 24 hours of onset. These selected participants were given a face mask used to inhale 10L/min oxygen for 4 hours in the EVT+NBHO group, while the EVT group was given room air. It was found that the EVT+NBHO group significantly reduced infarct volume, improved their NIHSS score in 7 days, and increased rates of functional independence at 90 days compared with the control group [13]. After this study, a multicenter large-scale RCT-OPENS-2 study has been carried out for interim analysis. The project is intended to include 280 patients with anterior circulation macrovascular occlusion given an intervention within 6 hours of onset. The intervention is the same as OPENS-1. The main endpoint of observation is the 90-day modified Rankin Scale (mRS).

Taking this into consideration, along with the current results, we see that NBHO should be started as early as possible, even before vascular recanalization, to achieve the best results and continued for a reasonable period, maybe 1-2 hours, to achieve "penumbra frozen" [14]. In the future, NBHO treatment may even start in the ambulance (Table 1).

## Protective Mechanism of NBHO

Although intravenous thrombolysis and endovascular thrombectomy have been verified to be effective, recanalization after cerebral ischemia often still leads to reperfusion injury, which may cause dysfunction of the BBB. Additionally, no efficient therapy can be administered before recanalization. Based on many animal studies, two major mechanisms have been emphasized as follows.

### NBHO increases penumbra oxygen supply to improve brain metabolism.

**Figure 1.**
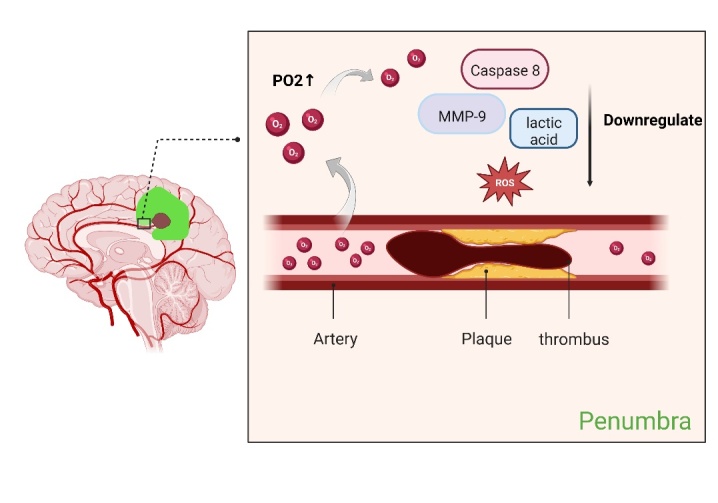
NBHO improves the PO2 in penumbra. When vascular was occluded, NBHO can increases penumbra oxygen supply to downregulate MMP-9, Caspase 8, lactic acid and ROS (Created with BioRender.com).

Acute ischemic stroke occurs due to vessel occlusion which leads to low oxygen delivery to tissues. Liu et al. conducted a study which found that maintaining penumbral PO_2_ at the pre-ischemic level by administering 95% O_2_ during ischemia resulted in a smaller volume of cerebral infarction in the hyperoxia group compared to the normoxic group. Additionally, the neurological function of the hyperoxia group was considered improved. Further research has shown that improving penumbra oxygenation leads to a decrease in the production of reactive oxygen species (ROS), matrix metalloproteinase-9 (MMP-9), and caspase-8 in the penumbra, providing neuroprotection [15, 16]. This improvement significantly ameliorates the effects of the no-reflow phenomenon by addressing the production of ROS (Fig.1).

### NBHO protects the blood-brain barrier and expands the time window of thrombolysis.

**Figure 2.**
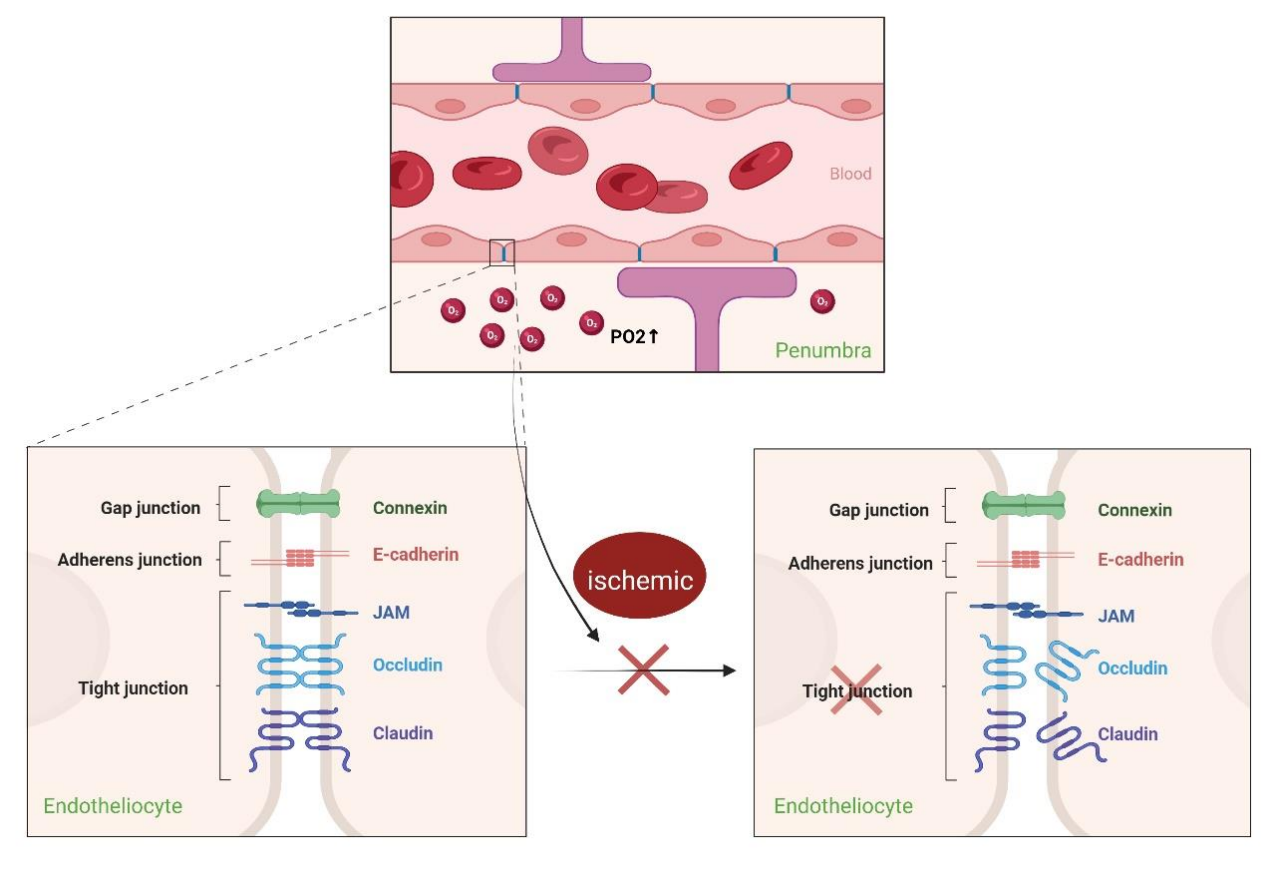
NBHO protects blood-brain-barrier. Tight junction is broken during ischemic, while NBHO can slow its progression (Created with BioRender.com).

Thrombolysis and mechanical thrombectomy are time-restricted treatments [17, 18]. The degree of blood-brain barrier (BBB) disruption is one of the major factors that impacts the prognosis of thrombolysis. A previous study showed that early use of NBHO can reduce BBB damage and significantly improve the outcome of delayed tPA treatment. This means that NBHO could be an effective adjuvant to extend the time window of thrombolysis in rtPA. The possible mechanism for this may be that NBHO slows MMP-9 Induction and tight junction protein (TJP) loss [19]. In another experiment, Shi et. al. demonstrated that cerebral ischemia-reperfusion can lead to the degradation of cerebral microvascular occludins and claudin-5s in rats, resulting in the increase of BBB permeability, whereas NBHO could block the process entirely [9] (Fig. 2).

## Box-1 Clinically ineffective reperfusion (CIR)

CIR refers to an unfavorable clinical outcome despite "near complete" recanalization (mTICI2b-3) [20]. CIR may result from extensive brain tissue damage prior to EVT, cerebral edema, reperfusion injury, and the no-reflow phenomenon. Therefore, the indications for NBHO include further improving the prognosis of patients based on recanalization, as mentioned previously. NBHO can protect the BBB, reduce cerebral edema and reperfusion injury, and improve reperfusion overall. Regarding the no-reflow phenomenon, oxygen can directly diffuse to hypoxic brain tissue, independent of complete blood flow recovery, which buys more time for postoperative blood flow. Whether NBHO can reduce the occurrence of no-reflow needs confirmation in clinical trials. CTP imaging was performed before and after thrombectomy to assess whether the NBHO group could improve blood flow after recanalization.

## Conclusion

Overall, NBHO appears to be a promising neuroprotective strategy that can assist vascular recanalization and improve patient outcomes. The mechanisms underlying penumbra freezing and BBB protection have been shown to protect the BBB,reduce cerebral edema and reperfusion injury, all of which can contribute to better reperfusion. To explore its potential for wider use, perfusion scans can be used to identify patients who are beyond the standard time window for reperfusion therapy but still meet the indications for treatment. By administering NBHO before reperfusion therapy, patients may experience even better protective effects and a more favorable prognosis. In fact, NBHO may even be administered in the ambulance to prolong the existence of the penumbra, thereby allowing more AIS patients to benefit from recanalization therapy and reducing the risk of mortality and disability.
